# Nitric Oxide Synthase Promotes Distension-Induced Tracheal Venular Leukocyte Adherence

**DOI:** 10.1371/journal.pone.0106092

**Published:** 2014-09-02

**Authors:** Aigul Moldobaeva, Otgonchimeg Rentsendorj, John Jenkins, Elizabeth M. Wagner

**Affiliations:** Department of Medicine, Johns Hopkins University, Baltimore, Maryland, United States of America; Emory University, United States of America

## Abstract

The process of leukocyte recruitment to the airways in real time has not been extensively studied, yet airway inflammation persists as a major contributor to lung pathology. We showed previously *in vivo*, that neutrophils are recruited acutely to the large airways after periods of airway distension imposed by the application of positive end-expiratory pressure (PEEP). Given extensive literature implicating products of nitric oxide synthase (NOS) in lung injury after ventilatory over-distension, we questioned whether similar mechanisms exist in airway post-capillary venules. Yet, endothelial nitric oxide has been shown to be largely anti-inflammatory in other systemic venules. Using intravital microscopy to visualize post-capillary tracheal venules in anesthetized, ventilated mice, the number of adherent leukocytes was significantly decreased in eNOS^-/-^ mice under baseline conditions (2±1 cell/60 min observation) vs wild type (WT) C57BL/6 mice (7±2 cells). After exposure to PEEP (8 cmH_2_O for 1 min; 5 times), adherent cells increased significantly (29±5 cells) in WT mice while eNOS^-/-^ mice demonstrated a significantly decreased number of adherent cells (11±4 cells) after PEEP. A similar response was seen when thrombin was used as the pro-inflammatory stimulus. In addition, mouse tracheal venular endothelial cells studied *in vitro* after exposure to cyclic stretch (18% elongation) or thrombin both demonstrated increased p-selectin expression that was significantly attenuated by N^G^-nitro-L-arginine methyl ester, N-acetylcysteine amide (NACA) and excess BH_4_. *In vivo* treatment with the ROS inhibitor NACA or co-factor BH_4_ abolished completely the PEEP-induced leukocyte adherence. These results suggest that pro-inflammatory stimuli cause leukocyte recruitment to tracheal endothelium in part due to eNOS uncoupling.

## Introduction

Lung parenchymal injury induced by ventilatory over-distension has been studied for many years. Increased endothelial and epithelial barrier permeability and neutrophil recruitment provide the nexus of lung pathology [1]. Numerous studies have shown that products of nitric oxide synthases within alveolar epithelium [2] and pulmonary microvascular endothelium [3], [4], [5] can contribute to subsequent injury through signaling pathways that promote increased permeability and neutrophil sequestration. One presumed mechanism for this sequence of events is nitric oxide synthase (NOS) uncoupling leading to injurious superoxide production [6]. In general, the process of leukocyte recruitment specifically to large airways has not been extensively studied. Nickles and colleagues showed recently that tracheal distension due to ventilation at high tidal volumes caused release of early response inflammatory cytokines [7]. Past work from our laboratory demonstrated that neutrophil recruitment to the airway vasculature was enhanced during excessive ventilatory stretch induced by the application of positive end-expiratory pressure (PEEP) [8], [9]. The airway vasculature is a systemic vascular bed and as such, the site of leukocyte rolling and adhesion is within post-capillary venules [10]. Previous work demonstrates that neutrophil recruitment specifically is dependent on increased p-selectin expression within the post-capillary venules [9]. In general, systemic venular endothelial cells are characterized by an abundance of Weibel-Palade bodies, known reservoirs for von Willibrand factor, p-selectin, and several other inflammatory peptides [11]. Interestingly, Weibel-Palade body secretion is regulated by endothelial nitric oxide [11], [12]. Specifically, shear-induced release of endothelial-derived nitric oxide (NO) has been shown to limit Weibel-Palade body release of p-selectin [13]. NO has been shown to inhibit thrombin-induced exocytosis of Weibel-Palade bodies from endothelial cells by inhibiting N-ethylmaleimide sensitive factor, a protein that regulates Weibel-Palade body membrane fusion events necessary for exocytosis [14]. Thus the role *in vivo* of endothelial cell-derived NO in the airway vasculature during airway distension is not clear. In the present study we questioned whether the products of endothelial NOS (eNOS, NOS-3) contribute to or attenuate neutrophil recruitment during excessive airway distension imposed through the application of PEEP and compared it to the response of the pro-inflammatory protein thrombin. Because NO is known to attenuate neutrophil recruitment in systemic vascular beds, we hypothesized that neutrophil recruitment would be increased in eNOS deficient mice. Contrary to this hypothesis, results demonstrated that products of nitric oxide synthase in post-capillary venules of airways are pro-inflammatory.

## Methods

### Mice

Our *in vivo* protocol was approved by the Johns Hopkins Animal Care and Use Committee. Mice were fed standard laboratory chow (Teklad Global, Harlan Labs, Madison, WI) prior to study. Eight-week old male mice (20–26 g; C57BL/6: Charles River Wilmington, MA and eNOS^-/-^ on a C57BL/6 background: Jackson Labs, Bar Harbor, ME) were anesthetized with a ketamine/acepromazine mixture (10∶1 at 1.0 µl/g body wt; ip), intubated (18 gauge intracatheter) and ventilated (HSE-HA MiniVent, Harvard Apparatus; Holliston MA) at 120 breaths per minute (0.2 ml/breath). Mice were used either to study leukocyte recruitment by intravital microscopy or sacrificed for tissue harvest. A total of 45 mice were studied.

### Intravital microscopy

Intravital microscopy of tracheal post-capillary venules was performed using procedures previously described [15]. Briefly, the trachea was exposed and continuously superfused with warmed (37°C), Krebs buffer. A single post-capillary venule was selected in each mouse just beyond the end of the endotracheal tube, visualized for up to 2 hours, and the images were recorded. Leukocyte trafficking was quantified off-line by measuring: 1) leukocyte rolling velocity and 2) the number of adherent cells in a 200 µm length of vessel during a 5 min recording per time point (0, 20, 40, 60 min) after the application of positive end-expiratory pressure (PEEP) or thrombin challenge. Rolling leukocytes were defined as leukocytes that moved at a velocity less than that of erythrocytes in a given vessel. The rolling velocity of 10 leukocytes entering the vessel was determined by measuring the time required for a cell to move 50 µm along the endothelial wall and the 10 values were averaged for each time point studied. A leukocyte was defined as adherent to venular endothelium if it remained stationary for ≥30 s. Adhesion data is presented as the sum of adherent cells 0, 20, 40, and 60 min after PEEP or thrombin challenge.

### *In vivo* treatments

The effects of airway distension were evaluated by applying a 1 min period of 8 cmH_2_O of positive end-expiratory pressure (PEEP), 5 times with a 10 min interval of no PEEP between each PEEP application. This is the same protocol applied previously which demonstrated an increase in leukocyte recruitment during and after this treatment [15]. In separate experiments thrombin (0.01–0.5 Units/ml; 10 ml volume) was delivered by superfusing the trachea for 2 min. Increasing doses of the NO donor nonoate (0–1000 µM) were superfused over tracheal post-capillary venules in eNOS^-/-^ mice. In some experiments, to maintain eNOS dimerization, the NOS co-factor BH_4_ (5,6,7,8-tetrahydroboipterin; Sigma) or negative control NH_4_ (Schircks Laboratories, Switzerland) was superfused over the trachea for the duration of the experiment. In a final series, the antioxidant N-acetylcysteine amide (NACA or PBS vehicle control) was delivered 2 h prior to study (5 mg/mouse ip; [16]).

### Tracheal venular endothelial cell (TvEC) isolation and distension

As previously reported, tracheal segments devoid of large veins from C57BL/6 mice were dissected, minced and digested [9]. The cellular digest was filtered and centrifuged, the cell pellet was resuspended and placed on a gelatin-coated 35 mm dish. After 5–7 days, cells positively labeled with fluorescent Dil-ac-LDL (Molecular Probes, Eugene, OR) as an endothelial cell marker and labeled EphB4 (Santa-Cruz, Santa Cruz, CA) as a marker of venous endothelial cells were selected using the FACSAria cell-sorting system (BD Biosciences, San Jose, CA) and replated (0.2% gelatin-coated T-25 flask). Depending on outcome variable and prior to challenge, TvEC were incubated (2% FBS DMEM; 2 h) and treated with vehicle, N^G^-nitro-L-arginine methyl ester (L-NAME; 10^−5^ M), N-acetylcysteine amide (NACA; 5×10^−3^ M), superoxide dismutase (SOD; 10^−5^ M) or excess BH_4_ (10^−4^ M [17] for 30 min prior to challenge, TvEC were distended with the Flexercell tension Plus System (FX-4000T; Flexcell Int, Hillsborough, NC) using 5% elongation representing physiological levels of ventilatory stretch, 18% elongation representing pathophysiological overdistension [9], [18] or treated with thrombin (5 Units/ml) all for 5 min exposures.

### eNOS expression and phosphorylation status

After the *in vitro* distension protocol, eNOS expression and phosphorylation status were determined by Western blot analysis. Cell lysates were fractionated by SDS-PAGE and detected by Western blotting using mouse anti-eNOS (pS1177) monoclonal antibody (BD Transduction Laboratories, San Jose, CA) and anti-eNOS polyclonal antibody (Cell Signaling, Danvers, MA). Relative band intensities were quantified by densitometry using Un-Scan-It Gel software (Bio-Medicine, Orem, UT). Mouse beta-actin was detected on immunoblots as a loading control. Experiments were performed in triplicate.

### Nitric oxide determination after cyclic distension

Supernatants from cells exposed to 5% and 18% distension without/with treatments were collected and nitric oxide was determined directly using the Griess reagent assay (Molecular probes, Eugene, OR). Nitrite (µM) formed by spontaneous oxidation of NO was measured in triplicate experiments.

### Reactive oxygen species release after cyclic distension

Confluent TvEC were incubated in 2% FBS DMEM media for 2 h, with/without treatments, and DHE (dihydroethidium, a lipophilic cell-permeable dye that undergoes oxidation to ethidium in the presence of superoxide, hydrogen peroxide and hydroxyl radicals; 10 µM) or 2,7-dichlorodihydrofluorescein diacetate (H_2_DCF-DA; 10 µM) for 30 min. Cells were stimulated by 5% and 18% cyclic distension for 5 min. For DHE staining, cells were washed and fixed with 4% paraformaldehyde. Flexible well bottoms were removed with a scalpel and placed on a microscope slide. Fluorescent images were obtained with a microscope (Olympus IX-51) using a Sensicam High Performance camera (Cooke Corporation, Auburn Hills, MI) at 100× magnification (10× objective).

The non-fluorescent H_2_DCF-DA, which converts to highly fluorescent DCF (dichlorofluorescein) in the presence of ROS, was used in separate cell distension protocols. After distension, the cells were washed, detached by trypsinization, and fluorescence intensity was measured using a F-2500 fluorescence spectrophotometer (DIGILAB, Hitachi, Tokyo, Japan).

### Tracheal venular endothelial cell adhesion molecule expression

After the *in vitro* distension protocol or thrombin challenge, adhesion molecules were evaluated using flow cytometry and immunostaining. Staining reagents for adhesion molecules included: LIVE/DEAD Fixable Blue fluorescent reactive dye (Invitrogen, Life Technologies, Carlsbad, California), FITC labeled anti-p-selectin, PerCP Cy5.5-labeled anti-VCAM-1, PE labeled anti-e-selectin, APC labeled anti-CD31 (BD Biosciences). Mouse isotype IgG served as a negative control and only live cells were analyzed. Flow cytometry was performed with a FACSAria (BD Biosciences), and analyzed with FlowJo software (Tree Star, Ashland, OR). For immunohistochemistry, tracheal venular endothelial cells plated on 35 mm iBidi dishes (IBIDI Gmbh, Munich, Germany) were subjected to staining with FITC labeled p-selectin and counterstained with DAPI. After treatments, cells were fixed, blocked and incubated with FITC labeled p-selectin antibody (1 hr at room temperature). Cells were washed and mounted with Prolong Gold antifade reagent with DAPI. Images were obtained using an Olympus IX51 microscope.

### Statistics

All data are presented as the mean ± the standard error. A square root transform of count data was made and leukocyte adhesion was evaluated by unpaired t-tests or one-way ANOVA. Relevant within group comparisons were made using Dunnett's Multiple Comparison test. A square root transform of pixel densities and mean fluorescence data was made followed by repeated measures ANOVA or t-tests. A p value ≤0.05 was accepted as significant.

## Results

### Leukocyte recruitment after distension

**Figure 1:**
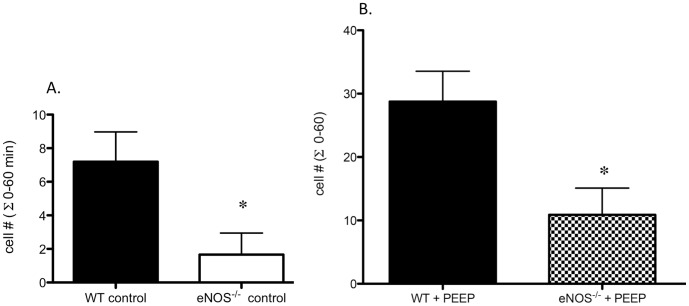
Leukocyte adherence in wild type (WT) and eNOS deficient (eNOS^-/-^) mice during control conditions and after the application of PEEP (positive end-expiratory pressure). **A.** The number of leukocytes adhered to tracheal post-capillary venules was summed over the course of 1 hr from measurements at 0, 20, 40, and 60 min (Σ 0–60). eNOS^-/-^ mice show decreased adherence during control experimental conditions (n = 5−6 mice/group); *p = 0.015). **B.** Effects of PEEP on tracheal venular leukocyte recruitment in wild type (WT), and eNOS^-/-^ mice. PEEP causes significant leukocyte recruitment as measured by the increased number of adherent cells. This increase was significantly reduced in eNOS^-/-^ mice. (* p<0.05 vs PEEP, n = 4−7 mice/group).

Using intravital microscopy to visualize post-capillary tracheal venules in anesthetized, ventilated mice, the number of adherent leukocytes was first evaluated under baseline conditions in wild type (n = 5) and eNOS^-/-^ deficient (n = 6) mice. **Figure 1A** shows the sum of adherent cells over the course of a 60 min experimental time period. eNOS^-/-^ mice showed a significantly decreased number of adherent cells compared to WT mice (p = 0.015). In a subsequent series of experiments, the effects of airway distension by the application of positive end-expiratory pressure (PEEP) were confirmed. As previously shown [15] and demonstrated in **Figure 1B**, distension induced by the application of intermittent PEEP caused significant leukocyte recruitment as measured by the sum of adherent cells compared to WT controls (p = 0.002). The number of adherent cells was significantly reduced when intermittent PEEP was applied in eNOS^-/-^ mice (* p<0.05 vs PEEP, n = 4−7/group). When evaluating leukocyte velocity in these experiments, only the application of PEEP in wild type (WT) mice had a sustained, significant (p<0.05; bold numbers) effect compared to control mice (**Table 1**).

**Table 1:** Leukocyte velocities.

TIME→	Baseline	0	20	40	60
TREATMENT↓					
**Control (WT)**	35	37	35	36	38
**PEEP**	37	**27**	**23**	**25**	**22**
**Thrombin (0.05)**	33	35	32	29	29
**eNOS^-/-^**	44	44	40	38	38
**eNOS^-/-^ + PEEP**	40	36	30	29	28
**eNOS^-/-^ + thrombin**	38	35	34	35	36
**PBS + PEEP**	34	32	**27**	**23**	**20**
**NACA + PEEP**	32	34	35	30	32
**NH_4_ + PEEP**	30	**24**	**22**	**20**	**24**
**BH_4_ + PEEP**	42	33	33	33	33

Mean values for leukocyte velocity (µm/sec) for the major study groups (SE ranged from 0 to 5µm/sec). Only the application of PEEP in wild type (WT) or vehicle (PBS and NH_4_) treated mice had a sustained, significant (p<0.05; **bold numbers**) effect on leukocyte velocity compared to control mice.

In a subsequent series of experiments in eNOS^-/-^ mice (n = 5), an attempt was made to add back NO to post-capillary venules *in vivo*. Increasing doses of the NO donor nonoate (0–1000 µM) were superfused over tracheal post-capillary venules. Only at the very highest concentration (1000 µM) was there a small increase (3–5 cells post treatment) in the total number of adherent cells.

### Leukocyte recruitment after thrombin

**Figure 2:**
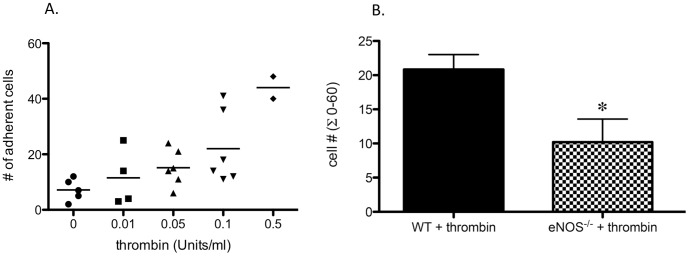
Effects of thrombin on tracheal venular leukocyte adherence *in vivo*. **A.** To determine the effects of thrombin on tracheal venular leukocyte adherence, superfused thrombin (2 min exposure) was assessed. A clear dose dependency was observed (n = 6 WT mice). **B.** Using a thrombin concentration (0.05 Units/ml) shown to cause a similar degree of cell adherence as the PEEP protocol, recruitment was significantly greater in WT compared to *eNOS^-/-^* mice. (* p<0.05; # p<0.01; n = 5−6 mice/group).

To determine whether the NOS-induced difference in the response to distension was unique to this physical stimulus or whether it was a more general phenomenon, an inflammatory agonist known to cause leukocyte recruitment in other vascular beds was studied. Superfusion of thrombin showed a clear dose dependency in causing leukocyte adherence in tracheal post-capillary venules (**Figure 2A**; n = 6 mice). Based on this dose-response data, a thrombin concentration (0.05 Units/ml) was selected that caused a similar degree of cell adherence as the PEEP protocol, and was studied in WT mice and eNOS^-/-^ mice. eNOS^-/-^ mice showed a significant reduction in the number of adherent cells compared to wild-type mice treated with thrombin (**Figure 2B**; p<0.05; n = 5−6 mice/group). Thrombin had no effect on leukocyte velocities (Table 1).

### eNOS expression and phosphorylation status

**Figure 3:**
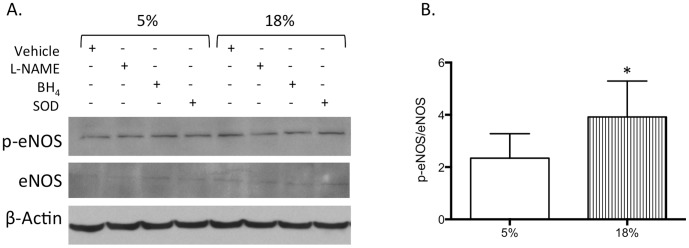
eNOS phosphorylation in tracheal venular endothelium following distension. **A.** Representative Western blot showing eNOS phosphorylation (p-eNOS) in mouse tracheal venular endothelial cells exposed to 5% vs 18% cyclic distension, without (Vehicle) and with eNOS inhibitor L-NAME, co-factor BH_4_, and anti-oxidant superoxide dismutase (SOD). Only 18% distension showed a reproducible increase in p-eNOS expression. Results are representative of n = 3 experiments). **B.** Quantification of p-eNOS expression by densitometry and normalized to total eNOS protein expression (p-eNOS/eNOS). Expression after 18% distension was significantly increased (* indicates p<0.05).

Cells were uniformly challenged *in vitro* to determine whether cyclic elongation of mouse tracheal venular endothelial cells showed enhanced eNOS phosphorylation when excessive distension (18%) was applied compared to a physiologic level of distension (5%). **Figure 3A** is an image of a representative Western blot showing eNOS phosphorylation (p-eNOS; pS1177) in mouse tracheal venular endothelial cells exposed to 5% vs 18% cyclic distension, without (vehicle) and with eNOS inhibitor L-NAME, co-factor BH_4_, and anti-oxidant superoxide dismutase (SOD). Only 18% distension showed a reproducible increase in p-eNOS expression. Quantification of p-eNOS expression by densitometry and normalized to total eNOS protein expression (p-eNOS/eNOS) showed a significant increase in cells exposed to 18% compared to 5% distension (**Figure 3B**, p<0.05; n = 3 experiments). Cells exposed to 18% and treated with pharmacologic inhibitors showed no difference in p-eNOS expression from paired 5% distended, treated cells.

### Nitric oxide determination after cyclic distension

**Figure 4:**
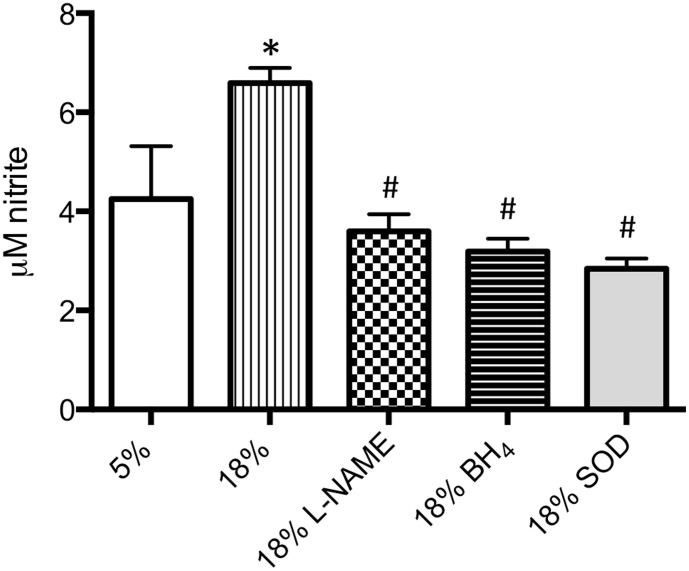
Nitric oxide release following cyclic distension. Nitrite (µM) formed by spontaneous oxidation of NO was measured with physiological cell distension (5%) and after excessive distension (18%) without and with eNOS inhibition (L-NAME), excess co-factor (BH_4_), and with the anti-oxidant superoxide dismutase (SOD). A significant increase in nitrite was seen in cells exposed to excessive distension compared to 5% distension (* indicates p<0.05). Each of the 3 inhibitors significantly decreased the level of nitrite in cells exposed to 18% distension (# indicates p<0.01; n = 3 experiments/group).

Nitrite (µM) formed by spontaneous oxidation of NO was measured with physiological cell distension (5%) and after excessive distension (18%) without and with eNOS inhibition (L-NAME), excess co-factor (BH_4_), and with the anti-oxidant superoxide dismutase (SOD). A significant increase in nitrite was seen in cells exposed to excessive distension compared to 5% distension (**Figure 4**; p<0.05). Each of the 3 inhibitors significantly decreased the level of nitrite in cells exposed to 18% distension (p<0.01; n = 3 experiments/group).

### Reactive oxygen species release after cyclic distension

**Figure 5:**
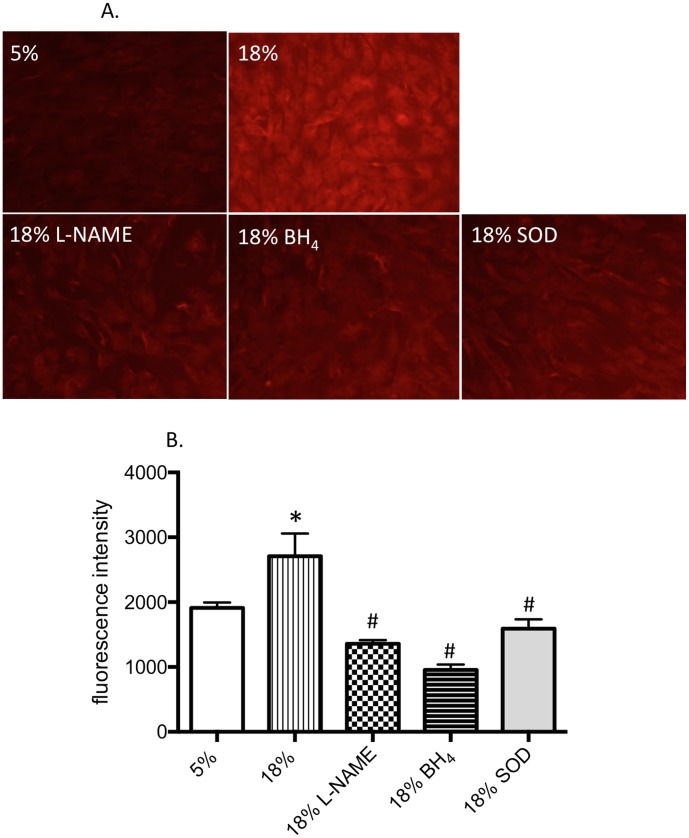
ROS release following cyclic distension *in vitro*. **A.** Tracheal venular endothelial cells evaluated for dihyrdoethidium (DHE; red) uptake after 5% and 18% distension, with inhibitors. Visualization of ROS expression shows a clear increase in ROS after 18% distension compared to cells exposed to 5% distension. Treatment of cells with the pharmacological inhibitors reduced DHE fluorescence in these representative images. **B.** Quantification of ROS release by dichlorofluorescein. Tracheal venular endothelial cells exposed to 18% distension showed a significant increase in fluorescence intensity compared to cells exposed to 5% distension (*indicates p<0.05). Furthermore, each of the pharmacological treatments significantly decreased ROS expression in cells exposed to 18% distension (# indicates p<0.01 vs 18%; n = 3 experiments/group).

To evaluate reactive oxygen species release by excessively distended tracheal venular endothelium, two strategies were taken. Mouse TvEC were loaded with the dye dihyrdoethidium (DHE) that fluoresces upon ROS exposure and were exposed to 5% distension, 18% distension, and 18% distension plus inhibitors. Visualization of ROS expression shows a clear increase in endothelial cell ROS after 18% distension compared to cells exposed to 5% distension (**Figure 5A**). Treatment of cells with the pharmacological inhibitors reduced DHE fluorescence in these representative images. In parallel, endothelial cell ROS was quantified using another fluorescent dye, dichlorofluorescein. TvEC exposed to 18% distension showed a significant increase in fluorescence intensity compared to cells exposed to 5% distension (**Figure 5B**; p<0.05). Furthermore, each of the pharmacological treatments significantly decreased ROS expression in cells exposed to 18% distension (p<0.01 vs 18%; n = 3 experiments/group).

### P-selectin expression in tracheal venular endothelial cells

**Figure 6:**
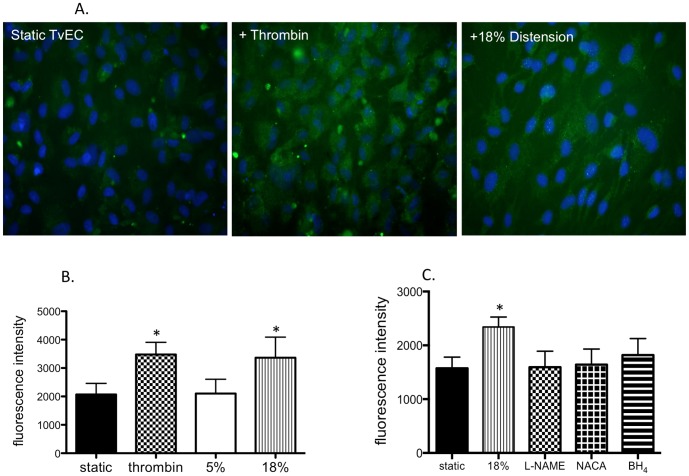
P-selectin expression following cyclic distension in tracheal venular endothelial cells. **A.** Fluorescence imaging of static TvEC (left panel), TvEC exposed to thrombin (center panel), and TvEC exposed to 18% distension (right panel). P-selectin expression (green fluorescence) indicates anti-p-selectin antibody and DAPI (blue) identifies cell nuclei. Both thrombin and 18% distension show enhanced p-selectin expression. **B.** P-selectin expression was quantified by flow cytometry (fluorescence intensity) in mouse tracheal post-capillary venular endothelial cells exposed to thrombin and 18% elongation. P-selectin expression was significantly increased after thrombin and 18% elongation relative to their respective controls (*p<0.05; n = 5 experiments/group). **C.** Effect of NOS inhibition (L-NAME), ROS inhibition (NACA), and excess eNOS co-factor BH_4_ on p-selectin expression during 18% distension. All inhibitors significantly reduced p-selectin expression in cyclically distended TvEC (*p<0.05; n = 4 experiments/group).

The effects of distension (18% elongation) and thrombin treatment were studied *in vitro* in TvEC. **Figure 6A** shows fluorescent anti-p-selectin expression of static tracheal venular endothelial cells (TvEC) compared with cells treated with thrombin and after 18% distension. An obvious increase in p-selectin expression (green fluorescence) in endothelial cells is seen after thrombin treatment. Endothelial cells exposed to distension show characteristic elongation but enhanced p-selectin staining. Quantification of these changes using flow cytometry showed that p-selectin expression (mean fluorescence intensity) was increased after thrombin treatment compared to vehicle treated, static cells (**Figure 6B**). Furthermore, cyclic elongation of tracheal venular endothelial cells showed increased p-selectin expression relative to cells exposed to a physiological level of elongation (5%; n = 5 experiments/group; p<0.05). No differences were seen in expression of E-selectin, VCAM, or CD31 when comparing static vs thrombin or 5% vs 18% elongation. In another series of experiments, treatments to block eNOS or ROS including L-NAME, NACA, and BH_4_ significantly decreased the p-selectin expression seen after 18% elongation (p<0.05; **Figure 6C**). Similar results were observed after inhibitors were used with thrombin treatment (p = 0.001; n = 4).

### ROS inhibition and BH_4_ superfusion block distension-induced leukocyte recruitment *in vivo*

**Figure 7:**
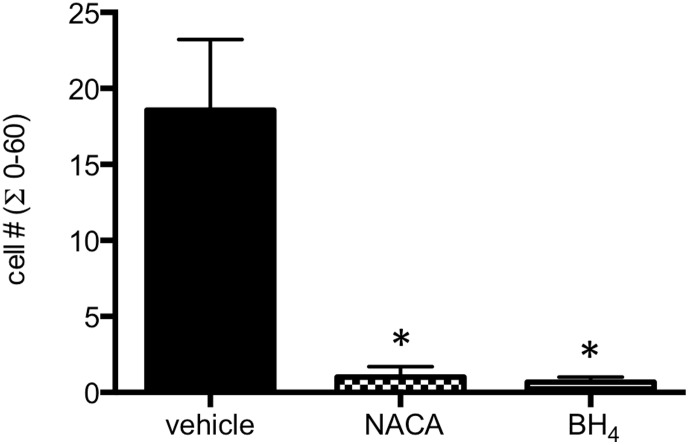
Effects of PEEP on tracheal venular leukocyte recruitment in wild type mice pre-treated with vehicle (PBS/NH_4_), anti-oxidant NACA, and eNOS co-factor BH_4_. Blocking reactive oxygen species *in vivo* with NACA or providing excess co-factor, completely blocked PEEP-induced leukocyte recruitment to tracheal post-capillary venules (* indicates p = 0.01 from vehicle; n = 3−4/group).

Given the *in vitro* results demonstrating a decrease in p-selectin expression with ROS inhibition and co-factor supplementation, the effects of ventilatory distension on leukocyte recruitment were studied without and with the potent anti-oxidant NACA and with the eNOS co-factor BH_4_ in WT mice. There were no differences in baseline adherence among the groups. Pre-treatment with NACA or superfusion with co-factor BH_4_ completely blocked the PEEP-induced increase in leukocyte adherence (**Figure 7**, p<0.01; n = 3−4/group). A comparable increase in adherent leukocytes in vehicle treated mice exposed to PEEP was seen as previously demonstrated in Figure 1B and leukocyte velocity (Table 1). Mice treated with NH_4_ did not differ from PBS vehicle treated mice so were included in the vehicle group. There were no changes in leukocyte velocity in NACA or BH_4_ treated mice.

## Discussion

The main finding of the current study is that endothelial nitric oxide synthase (eNOS, NOS-3) contributes to leukocyte recruitment to the airway when pro-inflammatory stimuli are imposed *in vivo*. Our previous work demonstrated that excessive ventilatory distension of tracheal post-capillary venules lead to increased p-selectin expression and neutrophil recruitment [9]. However, given the anti-inflammatory properties of endothelial cell-derived nitric oxide, the integrated *in vivo* response to ventilatory distension in eNOS deficient animals could not be predicted. Our experimental results demonstrated that both PEEP- and thrombin-induced leukocyte recruitment to the airways were reduced when eNOS activity was eliminated. *In vitro* studies of tracheal venular endothelium further confirmed that inhibiting nitric oxide synthase limited p-selectin expression. Importantly, anti-oxidant treatment of endothelial cells also limited both thrombin and distension-induced p-selectin expression. Since others have shown eNOS uncoupling results in superoxide release in the pulmonary circulation as well as other vascular beds, we interpreted these results as demonstrating that both distension and thrombin cause oxidant release and increased p-selectin expression. We confirmed this observation *in vivo* by demonstrating that blockade of ROS or delivery of excess co-factor BH_4_ completely eliminates distension-induced leukocyte recruitment.

The effects of NO on leukocyte recruitment in peripheral vascular beds have been known for many years in studies using intravital microscopy. Constitutive production and release of NO play a critical role in attenuating leukocyte adhesion. Experiments using non-specific inhibitors of NOS, in addition to work in eNOS^-/-^ mice have shown an increase in leukocyte adherence in post-capillary venules of the mesentery [19], heart [20], and cremaster muscle [21] under baseline conditions and in response to injurious stimuli. This effect was shown to be minimally related to changes in leukocyte velocity [19], and due primarily to an increase in p-selectin expression [22]. Delivery of exogenous NO could reverse these pro-inflammatory effects [23]. Lowenstein and colleagues showed in a series of studies that an increase in NO within post-capillary endothelial cells caused a decrease in Weibel Palade body exocytosis and consequently a decrease in p-selectin expression and subsequently, a decrease in leukocyte adhesion [13]. Based on this literature, we fully expected to see an increase in leukocyte adherence in eNOS^-/-^ mice after exposure to pro-inflammatory ventilatory distension and thrombin exposure.

Thus, our observation that baseline leukocyte adherence in tracheal post-capillary venules of eNOS^-/-^ mice was significantly reduced compared to wild type mice (Figure 1A) was unexpected as well as decreased leukocyte recruitment after PEEP (Figure 1B). We tested the specificity of the findings further by using another pro-inflammatory stimulus, thrombin, applied directly to the tracheal post-capillary venules. Thrombin has been shown to cause increased leukocyte recruitment in several beds [13], [24]. As expected, thrombin caused a predictable dose-dependent increase in leukocyte adhesion in post-capillary tracheal venules (Figure 2A). When using a dose of thrombin that resulted in a comparable increase in the number of adherent leukocytes as obtained with the application of PEEP, a similar reduction in the number of adherent cells was seen in the eNOS^-/-^ mice (Figure 2B). Although it is possible that the vector of endothelial cell distension with the application of PEEP was unique such that eNOS activation was pro-inflammatory, the application of thrombin via superfusion was similar to that done previously in other beds. Thus, both pro-inflammatory stimuli showed attenuated responses in the eNOS deficient mice, which were unexpected results compared to previous *in vivo* work by others in other organs. Although there was a sustained decrease in leukocyte velocity after the application of intermittent PEEP (Table 1), we showed previously that this change did not contribute to the changes in leukocyte recruitment [8]. Additionally, no changes in leukocyte velocity were seen after thrombin suggesting that changes in velocity were not the primary cause of the increase in leukocyte adherence. Finally, although eNOS^-/-^ mice showed decreased control baseline adherence compared to WT mice (Figure 1), we believe this did not impact the response to distension or thrombin, for within individual groups, there was no correlation between baseline adherence and the magnitude of the overall response.

We attempted to give back NO directly to the tracheal post-capillary venules in eNOS^-/-^ mice to elicit a gain of response, however, superfusion of nonoate was ineffective when applied to the adventitial side of the vasculature except at very high concentrations. We concluded that NO delivery specifically to the venular endothelium in this *in vivo* system may be difficult to achieve.

We suggest that in the oxygen-rich lung environment, the tracheal vasculature is more like the pulmonary circulation than peripheral systemic beds in regards to leukocyte recruitment. The effects of ventilator-induced injury and overdistension in the pulmonary vasculature have been extensively studied. Contradictory observations have been reported regarding the role of NOS isoforms in acute lung injury [25]. Kuebler and colleagues showed that circumferential stretch of pulmonary vascular endothelial cells activates the release of nitric oxide [5]. Recent reports demonstrate that lung injury following overdistension may be the result of eNOS uncoupling [2]. In healthy blood vessels, NO is continually being produced by eNOS. Coupling is essential for eNOS to function normally. eNOS couples L-arginine oxidation with O_2_ reduction to produce NO. However, if there are substrate (L-arginine) or co-factor (tetrahydrobiopterin; BH_4_) limitations, eNOS catalyzes the reduction of O_2_ to form superoxide anion [6]. This reactive oxygen species production by eNOS uncoupling can lead to endothelial cell dysfunction, leukocyte recruitment, and injury [3].

To begin to determine the mechanism responsible for increased leukocyte recruitment specifically to the airway vasculature, we isolated mouse tracheal venular endothelial cells to study *in vitro* responses due to the transient nature of NO and reactive oxygen radicals, and the limitations of *in vivo* microscopy to visualize these reactive substances. Using cyclic elongation of isolated mouse tracheal post-capillary venular endothelial cells, we compared 5% elongation estimated to be the physiological degree of distension occurring with normal ventilation to 18% elongation purported to be a pathological level of ventilatory load in some clinical settings [18], [26]. Since eNOS function is fundamentally modulated by protein phosphorylation (in particular pS1179), we confirmed increased eNOS phosphorylation with increased distension in our *in vitro* model. This observation is consistent with the work of several other investigators demonstrating phosphorylation of eNOS with cyclic distension [27], [28]. Additionally, we showed increased NO (nitrite) production in cells undergoing 18% distension and this increase could be blocked by eNOS inhibition with L-NAME (Figure 5A/B). Furthermore, 18% distension increased p-selectin expression and this increase was attenuated by L-NAME treatment (Figure 6B/C). These results corroborate the *in vivo* findings in eNOS deficient mice that tracheal venular endothelial-NOS is involved in leukocyte recruitment.

Furthermore, ROS release was increased with 18% distension vs 5% distension. As expected, an inhibition of superoxide with SOD reduced the level of ROS (Figure 5) and the anti-oxidant NACA reduced p-selectin expression *in vitro* as well as completely eliminated leukocyte recruitment *in vivo* (Figure 7). These observations suggest that the mechanism for enhanced recruitment was through endothelial cell production and release of ROS.

Because we first saw differences in leukocyte recruitment in eNOS deficient mice, we focused on eNOS-related mechanisms of ROS production; specifically whether eNOS uncoupling was responsible for enhanced ROS release and in subsequent leukocyte recruitment. *In vivo* observations that delivery of the eNOS co-factor BH_4_, continuously during the exposure to PEEP, eliminated leukocyte adhesion provides important evidence for eNOS uncoupling (Figure 7). Delivery of NH_4_, a chemical analogue of BH_4_, caused typical PEEP-induced leukocyte recruitment and did not differ from PBS vehicle control. Endothelial cells undergoing 18% distension and treated with BH_4_ also showed reduced levels of p-selectin (Figure 6C) and decreased ROS release (Figure 5), all consistent with eNOS uncoupling. However, BH_4_ and SOD treatment both showed decreased NO (nitrite) production (Figure 4) as well as no change in eNOS phosphorylation from cells exposed to 5% distension (Figure 3). Based on work of others and if uncoupling were the only mechanism operative, we expected nitrite levels to be increased in BH_4_ and SOD treated cells [29], [30]. Our results suggest additional control of eNOS perhaps through alteration of Akt/protein kinase B, which regulates eNOS phosphorylation [31]. Additionally, the kinetics of release of NO might vary with different pharmacological interventions and we applied the same timed protocol for all cells [27]. Further work describing upstream signaling involvement is beyond the scope of the current study. However, eNOS uncoupling partially explains enhanced superoxide/ROS production and subsequent leukocyte adherence. Furthermore, these results are overall consistent with a recent study by Su and colleagues who, using intravital microscopy of the cremaster muscle, showed that eNOS uncoupling was responsible for increased leukocyte recruitment and adhesion in a model of chronic hyperglycemia [17]. Treatment with supplemental BH_4_ reversed leukocyte recruitment, as in our study supplemental BH_4_ reduced p-selectin expression and leukocyte adhesion.

Mechanical ventilation in clinical settings can exert extreme changes in airway pressure. Changes in lung volume cause airway distension and pro-inflammatory sequelae [7]. We have shown previously increased leukocyte adherence after the application of positive end-expiratory pressure [8], [9]. In the present study, we have shown that PEEP-induced distension of tracheal post-capillary venular endothelium results in leukocyte adherence due to reactive oxygen species release. Distension of tracheal venular endothelium causes eNOS phosphorylation, increased NO (nitrite) and ROS release. eNOS uncoupling is at least partially responsible for increased ROS and enhanced leukocyte recruitment. As such, the systemic vasculature in the lung responds in a manner similar to the pulmonary vasculature and likely contributes to the overall inflammatory cell burden of the lung.
